# Preemptive Wild Boar Reduction: A Bridge *Not* Too Far in Effective Response to ASF Threat in a Protected Area Under High Anthropogenic Pressure

**DOI:** 10.3390/ani16010007

**Published:** 2025-12-19

**Authors:** Paweł Nasiadka, Maria Sobczuk, Wanda Olech, Michalina Gmaj, Daniel Klich

**Affiliations:** Department of Animal Genetics and Conservation, Warsaw University of Life Sciences, Ciszewskiego 8, 02-786 Warsaw, Poland

**Keywords:** wild boar, epidemiological threat, protected areas, control methods

## Abstract

In Kampinoski National Park (KNP), located in central Poland near the Warsaw agglomeration, the ASF epidemic lasted five years (2017–2021). The virus likely entered the park via wild boar migrating along a natural ecological corridor or through unintentional transmission by residents and tourists. Between 2014 and 2021, intensive monitoring and wild boar population reduction were implemented. The wild boar population, estimated at 11 individuals/km^2^ in 2013, began to be reduced from 2014 onward, when ASF was first detected in Poland. In 2018, at the peak of the epidemic, the density of wild boar dropped to about 0.51 individuals/km^2^—the lowest in the park’s history. Between 2017 and 2021, 408 ASF cases were recorded, mainly in dead wild boar, although age and sex structure analysis suggests that the actual mortality rate could have been up to about 50% higher. The early intervention—culling—which was controversial from the National Park’s perspective, appears to have played a key role in controlling the situation and likely in limiting the epidemic. At the same time, the limited organizational resources of KNP highlighted the need for proactive management and close institutional cooperation in protected areas subject to high anthropogenic pressure.

## Simple Summary

Protected natural areas near large cities face increasing challenges from human activity and emerging wildlife diseases. This study examines Kampinoski National Park in central Poland to illustrate how African swine fever spread in such an environment between 2017 and 2021. The virus most likely entered the area through wild boars migrating along ecological corridors or accidentally via visitors. Early and intensive efforts—monitoring and population reduction—helped contain the outbreak. In 2018, when ASF reached its peak and 375 infected wild boars were found dead, the wild boar population in the park was already at its lowest level in history due to earlier depopulation measures. Thus, the peak of the disease occurred when the population was at its minimum because of preventive culling. The actual mortality rate may have been even higher. The results highlight that effective action is possible when taken early. In this case, reducing the wild boar population likely helped significantly limit the spread of the disease. This study emphasizes the need for proactive and strong cooperation between different institutions to protect nature in areas exposed to high anthropogenic pressure and the risks of infectious diseases.

## 1. Introduction

Protected areas play a crucial role in conserving biodiversity, providing ecosystem services, and offering space for education and recreation [1,2,3]. Although their ecological and social value is undisputed, they are not immune to threats arising from human activities that may facilitate the spread of pathogens [4]. Human-borne pathogens, invasive species, and excessive tourism pressure can pose serious risks to the integrity of these areas [5]. In crisis situations, such as wildlife infectious disease outbreaks, national parks can be particularly vulnerable to organizational paralysis due to limited staffing, strict conservation regulations, and a lack of systemic support—more so than areas under more direct human control, such as commercial forests or agricultural landscapes [6]. National parks such as the KNP operate within a complex and often contradictory framework: on one hand, they provide relatively undisturbed habitats for wildlife; on the other hand, their popularity as tourist destinations may carry a risk of pathogen transmission from visitors. This risk may manifest in three ways: the introduction of pathogens into the park, their transmission within park boundaries, and their export beyond park boundaries. African swine fever (ASF) is a viral disease affecting suids, and while humans are not biological hosts, they can act as passive vectors, potentially introducing the virus through these pathways [7,8,9].

ASF is a highly contagious viral disease that affects domestic pigs and wild boar, characterized by an almost 100% mortality rate in infected animals [10]. Originally restricted to Africa, the ASF virus has spread to Europe, Asia, and other parts of the world, causing severe losses in livestock farming and disruption to forest ecosystems [11,12]. In Europe, wild boar populations and humans have become important in the passive spread of the virus, significantly complicating efforts to effectively eradicate the disease [11,13].

The first case of ASF in wild boar in Poland was recorded in 2014. Since then, the disease has spread across much of the country. Strict control measures were implemented in response, including wild boar population reduction, improved biosecurity in agriculture, forestry, and hunting, and changes to wildlife population management regulations [14]. A particular challenge has been reducing wild boar populations within protected areas, where such interventions are legally restricted and logistically difficult to implement. While population reduction in hunting grounds was relatively easy to organize, the situation in national parks proved much more complex.

National parks in Poland are established under the Nature Conservation Law, with the primary aim of preserving biodiversity and maintaining ecosystem functions in as natural a state as possible [15]. In contrast to hunting grounds, where wildlife populations are managed by hunters and foresters [16], population control measures in national parks are limited to exceptional circumstances, such as crop damage or threats to livestock. In national parks, population reduction is carried out by a limited number of staff and a small group of volunteer hunters, whereas in hunting grounds, these activities involve hundreds of hunters and foresters.

Kampinoski National Park (KNP) in central Poland is a unique example of how to address animal disease risks in protected areas. As one of the largest national parks in the country, located near the Warsaw metropolitan area with its approximately three million inhabitants, KNP is exposed to exceptionally high anthropogenic pressure. The park is also part of one of the most important ecological corridors in central Poland, stretching along an unregulated, natural section of the Vistula. At the same time, it is one of the most visited national parks in Poland, attracting around 1.5 million tourists annually [17]. As a typical lowland park, it is easily accessible and, due to its proximity to a large urban center, is used for recreational purposes year-round [18]. The consequences of such intense tourist traffic and proximity to a major city are manifold, ranging from wildlife disturbance and pollution to the risk of pathogen transmission within and beyond park boundaries [5,19,20].

The history of ASF in KNP shows that national parks—despite the constraints imposed by their legal status, organizational structure, and limited resources—need to consider the threat of wildlife diseases in their management strategies. As a case study, the ASF outbreak in KNP may provide useful insights for other protected areas facing similar challenges, while naturally remaining limited by the specific ecological and organizational context in which it occurred. Against this background, the present study sought to address three main objectives: (1) to document the spatial and temporal course of the ASF outbreak in Kampinoski National Park; (2) to examine the rationale and potential value of the early wild boar reduction measures undertaken; and (3) to estimate the potential scale of unfound wild boar carcasses.

## 2. Materials and Methods

### 2.1. Study Area

**Figure 1:**
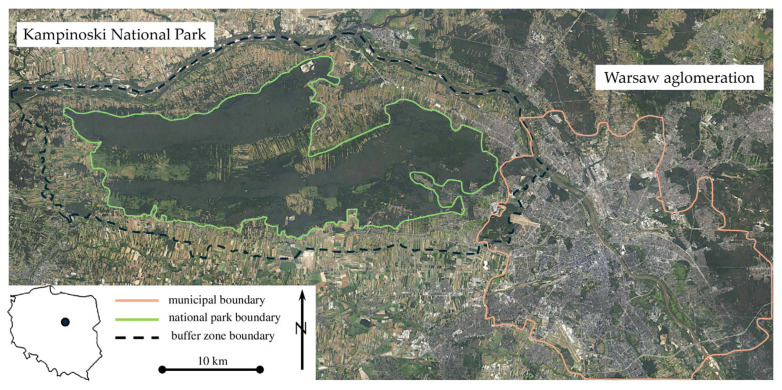
Location of the Kampinoski National Park and the Warsaw metropolitan area (Source: Google Maps, accessed on 2 June 2025).

The KNP is one of the largest national parks in Poland [21]. It is located in central Poland, on the northwestern border of the largest Polish city, Warsaw. Its location near the Vistula Valley makes it one of the most important elements of the Polish ecological network. The park was established in 1959, primarily to protect the unique complex of inland dunes and the rich diversity of flora and fauna [22]. In 2000, the KNP and its buffer zone were recognized by UNESCO as a biosphere reserve and included in the European ecological network NATURA 2000—PLC 140001 [23] (Figure 1).

The park covers an area of 385.44 km^2^, with a buffer zone of 377.56 km^2^ surrounding it. The largest part of KNP—about 73% of the area—is covered by forests (about 270 km^2^); the remaining area consists mainly of meadows, grasslands, peat bogs, and arable land. The forests are dominated by pine (*Pinus sylvestris*, 69%), black alder (*Alnus glutinosa*, 12.5%), oak (*Quercus* sp., 10.3%), and birch (*Betula* sp., 6.4%). Arable land covers about 2000 ha (5.3% of the KNP area) and is privately owned by residents of 39 villages that are wholly or partly located within the park. After the detection of ASF in Poland in 2014, pig farming in the vicinity was restricted to large, fully controlled, and isolated farms. There are no pig farms in the immediate vicinity of KNP, so domestic pigs do not pose a direct risk of ASF introduction into the park. Agriculture in the park is closely linked to the terrain. The arable land is spatially fragmented, consisting mainly of small patches of drained wetlands used for agriculture. The fields are small, close to farm buildings, and often border the woodlands managed by the park. Due to the poor soil quality, crops grown here are those that make few demands on the habitat, mainly rye and, in recent decades, maize.

The park’s proximity to Warsaw makes it one of the most important recreational areas for the approximately 3 million inhabitants of this metropolitan area. The park is accessible via 35 entrances and several transit routes. The total length of marked trails is more than 360 km, including 55 km of bicycle paths (plus 145 km in the vicinity of the park). The park has 15 forest parking lots and 6 green areas with recreational facilities. The park service estimates that around 1.5 million visitors come to the KNP every year.

Tourists and locals explore the park year-round. The KNP is completely surrounded by a highly urbanized landscape with a dense network of local roads and highways. In the south, these roads connect the towns of Ożarów Mazowiecki (approximately 20,000 inhabitants), Błonie (approximately 15,000 inhabitants), and Sochaczew (approximately 35,000 inhabitants) over a distance of about 25 km. To the east, the park borders Warsaw, while to the north it is bordered by the S7 highway for about 24 km and a dense urban corridor stretching for about 50 km. The park is not fenced, and wild boar can move freely across its administrative boundaries; however, the mosaic of wetlands, forests, roads, and built-up areas creates semi-permeable landscape units that influence the ease of movement and mixing between animals inside and outside the park. The northern border of the KNP buffer zone is Poland’s largest river, the Vistula, which remains unregulated in this section and serves as an important ecological corridor in central Poland. The river functions primarily as a linear movement corridor facilitating wildlife dispersal. During periods of high water, it may locally slow the movement of wild boar, but it does not constitute a permanent physical or epidemiological barrier.

From a faunistic perspective, the KNP is known for the successful reintroduction of moose (*Alces alces*) in 1951. The large ungulates living in the park also include red deer (*Cervus elaphus*), roe deer (*Capreolus capreolus*), and wild boar (*Sus scrofa*). The beaver (*Castor fiber*) was successfully reintroduced in 1999, and the lynx (*Lynx lynx*) in 1992. Since the early 2000s, two wolf packs (*Canis lupus*), totaling around 12–15 individuals, have been recorded in the KNP.

### 2.2. Data Collection

Following the first detection of ASF in Poland in 2014, a wild boar population monitoring program was initiated at the request of the national veterinary authorities. This program included the mandatory search for wild boar carcasses, which—together with hunted animals—were tested for the presence of the ASF virus.

In the KNP, park rangers continuously searched for wild boar carcasses during their daily patrols. When clusters of dead animals were found, additional collective searches were organized in the surrounding areas of potential outbreak foci.

Although the searches were not conducted according to a systematically planned protocol, the continuous monitoring by the rangers ensured the representativeness and reliability of the collected data.

Initially, only hunters and park rangers participated in the organized searches; however, as the epidemic progressed, local fire brigades and military units were also involved. During collective searches, participants systematically surveyed designated areas, moving in a line across strips of terrain ranging from several dozen to several hundred meters in width and extending up to several kilometers in length.

If a wild boar carcass or its remains were found, ASF testing was performed by the National Veterinary Institute in Puławy according to standard EU procedures. The sex and age of the animal were determined. Age was divided into three categories: piglets (up to 12 months old), yearlings (12–24 months old), and adults (over 24 months old). The locations of detected carcasses were recorded on digital maps using commonly available applications such as Google Maps, Geoportal^®^, and Forest Data Base^®^. These data facilitated the estimation of average linear distances between successive carcass discoveries and allowed analysis of the spatial spread of the epidemic over several years.

It is highly likely that the number of recorded wild boar carcasses underestimates the true extent of ASF-related mortality within the park. However, information on the sex and age of the deceased animals allowed for a more accurate characterization of the mortality pattern. To address representativeness, the age and sex structure of dead wild boars in the KNP was compared with historical data from three reference populations: the KNP population from the 1970s [24], wild boars from the experimental forests of the Poznań University of Life Sciences [25], and populations from southern Poland [26]. This comparison enabled adjustment for deviations in representativeness and provided reliable estimates of mortality across age classes. Such correction was possible for 2018 and 2019, when the numbers of carcasses recorded (63 in 2018; 266 in 2019) were sufficiently high to be considered representative of wild boar mortality in the KNP. Adult individuals were assumed to have the highest probability of being found, and their numbers were used as the basis for estimating mortality in the yearling and piglet categories. The likely actual number of wild boars that died as a result of ASF was estimated from the proportion of adults in the national populations, which averages about 22.7%. The proportions of the remaining age classes were estimated at about 28.4% for yearlings and about 48.9% for piglets (Table 1). It should be noted that although this correction likely results in a more realistic age-class distribution, the overall mortality estimates remain conservative. This is because the number of adult wild boars found—which forms the basis for subsequent calculations—may itself be underestimated, as not all carcasses are likely to have been detected. Therefore, the corrected values should be regarded as a minimum estimate of mortality.

In parallel with the search for wild boar carcasses resulting from ASF, depopulation measures were implemented both within the Park and across the wider Mazovia region. In the KNP, population reduction was carried out exclusively by a small number of field-based Park staff and formally designated volunteer hunters; no commercial hunting teams or military units were involved. The culling aimed solely to reduce population density, with no selection based on sex or age, and was conducted throughout the year because wild boar are not protected by any closed season.

Depopulation actions were also carried out in the region surrounding the KNP by local hunting clubs operating under national ASF control regulations; however, these activities were administratively independent of the Park. Although both types of operations occurred simultaneously, regional depopulation likely had no impact on wild boar density or disease dynamics within the Park. This is primarily because the Park, with its large area (several hundred km^2^) and distinct landscape structure—a vast, continuous forest complex—supports an ecologically independent wild boar population. Although the KNP lies within a recognized ecological corridor and wild boar movements across its boundaries do occur, the scale of this movement does not appear to meaningfully affect population dynamics within the Park, even if it may be relevant for pathogen introduction. Consequently, any potential effect of hunting activities conducted outside the Park, although not directly assessed, can reasonably be considered marginal.

### 2.3. Data Analysis

All statistical analyses were conducted in R version 4.4.3 [27]. Variables included age class (piglets, yearlings, adults), sex, linear distance between consecutive carcasses, and population density estimates. The workflow was fully reproducible and relied on open-source R packages: tidyverse version 1.3.0 for data wrangling and visualization [28], rstatix version 0.7.2 for inferential statistics [29], effectsize version 0.8.9 for effect-size estimation [30], and rstanarm version 2.32.1 with bayesplot for Bayesian modeling and sensitivity analysis [31,32]. Because the data did not meet assumptions of normality or homoscedasticity, non-parametric procedures were applied throughout. To test deviations from a 1:1 sex ratio, a chi-square goodness-of-fit test was used, and effect magnitude was expressed as the Phi coefficient (Φ) with 95% confidence intervals. Differences in age-class structure (piglets, yearlings, adults) between years and relative to historical ASF-free reference populations were evaluated using contingency chi-square tests with Cramér’s V as an effect-size measure (95% CI) [33]. For spatial data, representing linear distances between consecutively detected carcasses, a Kruskal–Wallis test assessed overall temporal differences (2017–2021) [34], followed by pairwise Wilcoxon rank-sum tests [35] with Benjamini–Hochberg correction for multiple comparisons [36].

To describe the magnitude and direction of group differences, two effect-size indices were reported with 95% confidence intervals: Cliff’s delta (δ) and the rank-biserial correlation [37,38]. Finally, to address the uncertainty in carcass detectability and estimate the likely number of undetected deaths, two complementary approaches were implemented: (1) a deterministic sensitivity analysis, varying the assumed detectability of adult carcasses between 0.6 and 1.0; and (2) a Bayesian hierarchical model, which incorporated prior uncertainty about detection probability (Beta distribution) and age-class proportions (Dirichlet prior) and was summarized by posterior means, medians, and 90% credible intervals [31,32].

All statistical significance was assessed at α = 0.05, but interpretation emphasized effect sizes and credible intervals rather than dichotomous thresholds.

## 3. Results

In 2013, a year before the ASF outbreak in Poland, the wild boar population in KNP and its buffer zone was estimated at a very high level of about 4200 individuals (11.0 individuals/km^2^), based on regular year-round observations conducted by park staff and hunters. More than 1100 animals lived in the park itself, and around 3200 in the surrounding hunting districts. Before the ASF pandemic, wild boar hunts in the park aimed to stabilize the population (preventing further growth) and reduce damage to agriculture. In 2014, when the first ASF cases were reported in Poland, the wild boar population in KNP was estimated at about 1100 individuals (2.85 individuals/km^2^), and 702 animals were culled (1.82 individuals/km^2^), corresponding to about 60% of the spring population. This proportion, expressed as the percentage of animals harvested relative to the estimated spring population size, is hereafter referred to as population exploitation. In response to government recommendations to combat ASF, culling intensity increased by around 70% in 2015–2016. A total of 1268 wild boars were culled in 2015 and 1155 in 2016. Although these figures were higher than the spring population (population exploitation levels: 117% in 2015; 108% in 2016), the population density remained stable (2.78–2.80 individuals/km^2^).

A markedly reduced wild boar population resulting from previous culling efforts was first recorded in spring 2017, when density dropped to 1.82 individuals/km^2^. In the same year, 1275 wild boars were culled (3.31 individuals/km^2^), the highest annual number ever documented in the park. As a consequence of these intensive reduction measures, the population reached its historical minimum in 2018, estimated at only 206 individuals (0.53 individuals/km^2^). Despite this low abundance, culling continued, and a further 450 individuals were removed that year, corresponding to more than 200% of the spring estimate. This sustained pressure kept the population density similarly low in 2019, when numbers reached 227 individuals (0.59 individuals/km^2^).

**Figure 2:**
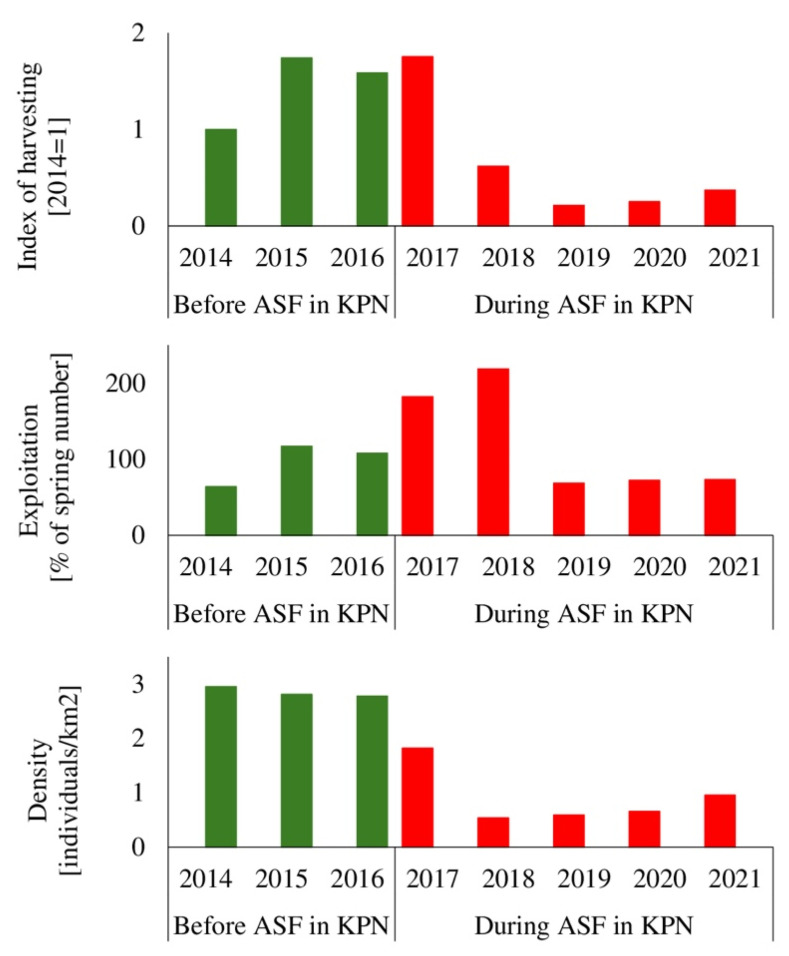
Wild boar harvest index, exploitation rate and density in Kapminoski National Park in the years 2014–2021.

After 2018, eradication became increasingly difficult due to the markedly reduced number of wild boars. In 2019–2021, annual harvests were only 20–30% of the 2014 level and less than 20% compared to the 2015–2017 depopulation period. The population exploitation rate reached 75% in 2020 and 73% in 2021. During this period, wild boar density gradually increased, reaching 0.65 individuals/km^2^ in 2020 and 0.95 individuals/km^2^ in 2021. Overall, these trends demonstrate an initial, steep population collapse during the early epidemic phase (2017–2018), followed by sustained suppression through 2019 and a gradual rebound once culling effort decreased, indicating a strong dependence of population dynamics on harvest intensity (Figure 2).

**Figure 3:**
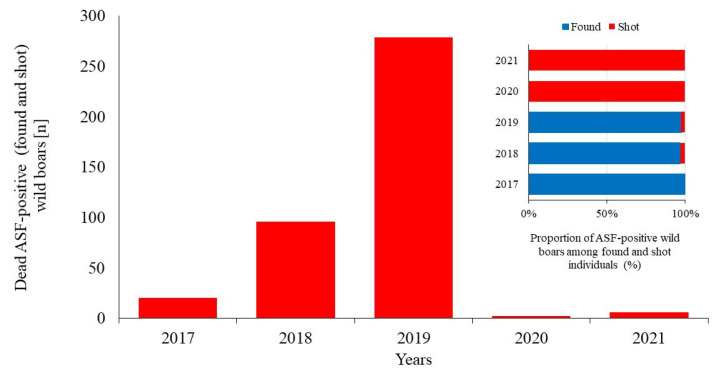
Numbers and share of found and shot of ASF positive wild boars in Kampinoski National Park in the years 2017–2021.

The ASF outbreak in KNP lasted five years and followed a highly dynamic course. The first 20 ASF-positive wild boars were confirmed in 2017, coinciding with the most intensive culling measures and marking the final phase of the depopulation campaign. All carcasses detected that year were ASF-positive, while none of the 1275 culled wild boars tested positive for the virus. In 2018, the number of infected wild boars rose to 96, including 3 culled animals that tested positive. In 2019, the estimated spring population was around 220 animals, of which 155 were culled, including 7 infected animals. That year, a total of 279 infected wild boars were confirmed: 272 were found dead and 7 were culled. In 2020 and 2021, no infected carcasses were found during field inspections. However, two cases (1.1%) were found among 182 culled wild boars in 2020, and six cases (2.2%) among 269 culled animals in 2021. No further cases of ASF were detected in the park or its buffer zone in 2022. In the first two years, a marked increase in ASF cases was recorded, followed by a substantial decrease; from 2020 onward, only sporadic detections were noted (Figure 3).

**Figure 4:**
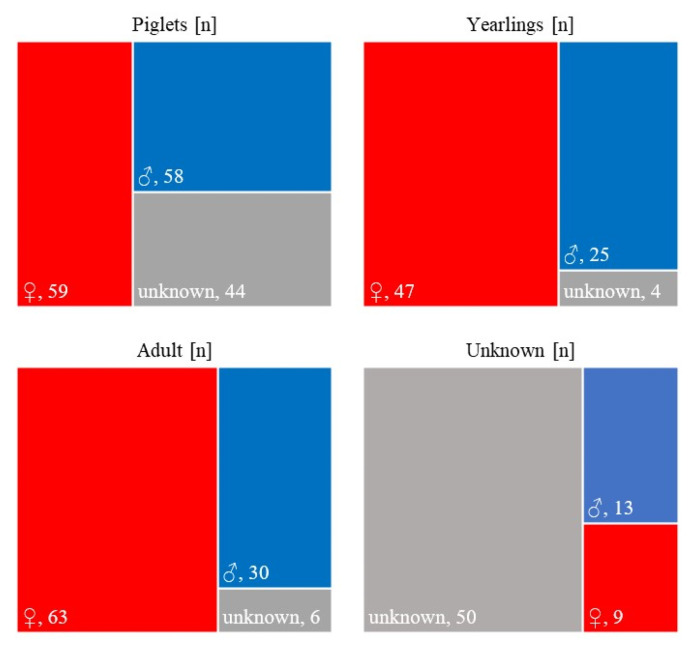
Numbers of males, females, and unclassified wild boars in four categories: piglets, yearlings, adults, and undetermined individuals among 408 ASF-infected wild boars recorded in Kampinoski National Park between 2017 and 2021.

The classification of ASF-infected wild boars into three age classes (piglets, yearlings, and adults) made it possible to assess the sex and age structure of ASF-related deaths and to compare these parameters with those of non-infected populations. Of 408 ASF-positive wild boars, both sex and age were determined in 336 animals (82.3%). This group included 161 piglets (47.9%), 76 yearlings (22.6%), and 99 adults (29.5%). Of the remaining 72 carcasses, the sex could only be determined for 22 animals (13 males and 9 females), while the remaining 50 could not be assigned to either sex or age. All analyses of sex ratios were conducted using only individuals with known sex; animals of undetermined sex were excluded from calculations. There were clear differences in the sex structure between age groups. In piglets, the proportions of males and females were similar (36.0% male and 36.6% female), with 27.3% undetermined. In contrast, females were identified more frequently in yearlings (61.8% female vs. 32.9% male, 5.3% indeterminate) and adults (63.6% female vs. 30.3% male, 6.1% indeterminate) (Figure 4).

Among all ASF-positive wild boars with known sex (n = 301), females (n = 179) significantly outnumbered males (n = 122) (χ^2^ = 10.79, df = 1, *p* = 0.001; Φ = 0.19, 95% CI [0.08, 0.30]). This represents a small-to-moderate effect size, indicating a detectable skew in the sex ratio toward females. When examined by year and age class, the age distribution of detected carcasses differed significantly from reference ASF-free populations. In 2018, the discrepancy was large (χ^2^(2) = 21.12, *p* < 0.001; Cramér’s V = 0.39, 95% CI [0.20, 0.57]), whereas in 2019 it was minor (χ^2^(2) = 6.05, *p* = 0.049; V = 0.11, 95% CI [0.02, 0.18]). These findings suggest that during the first epidemic peak, recovered carcasses were dominated by adults, while in the following year detection became more evenly distributed across age classes. Analysis of sex ratios within age classes showed no significant deviation from parity in 2018 (*p* > 0.1 for all classes), whereas in 2019 a marked female predominance was observed among adults (χ^2^ = 14.63, *p* < 0.001) and yearlings (χ^2^ = 9.62, *p* = 0.002), consistent with selective mortality or detection bias.

In 2018, the age of 63 wild boars was determined: 39.8% were piglets, 25.3% yearlings, and 34.9% adults. Assuming that all adult animals (n = 22) were found and converting the expected proportions of the remaining age classes, the actual number of piglets killed could have been around 50 animals—88% more than the number found. The number of dead yearlings was estimated to be around 30 animals, an increase of 75% over the actual number found. Overall, the estimated number of dead wild boars in 2018 could have amounted to around 100 individuals—about 50% more than the 63 carcasses found. Slightly smaller discrepancies were observed in the second ASF peak year, 2019. That year, the proportions of piglets, yearlings, and adults were 51.1%, 22.2%, and 26.7%, respectively. Based on the age class distribution in unaffected populations, the number of piglets that died was estimated at around 150—about 10% more than the 136 animals found. The number of dead yearlings was estimated at around 90, an increase of 50% over the 59 individuals recovered. Assuming that the carcasses of adult animals are fully recoverable, the total number of dead wild boars in 2019 could have been over 300—about 20% more than the 266 carcasses found in the field (Table 2).

**Figure 5:**
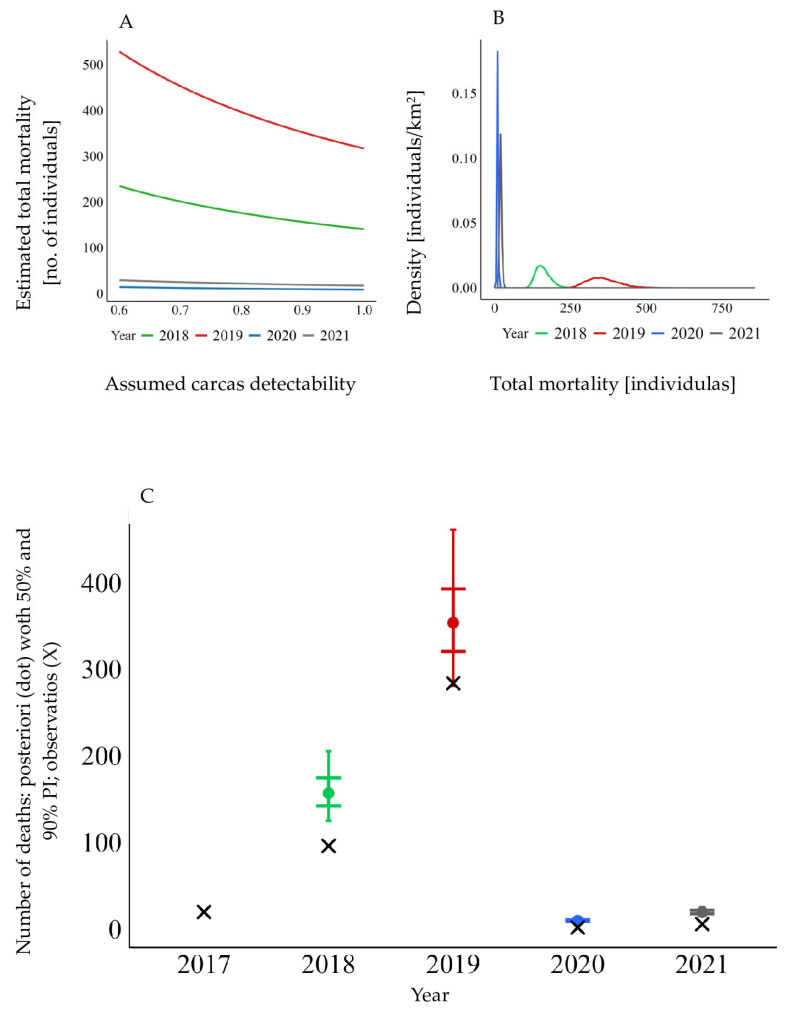
Sensitivity and Bayesian estimation of wild boar mortality: (**A**) sensitivity of total mortality estimates to the assumed carcass detectability; (**B**) posterior distributions of total mortality by year; (**C**) observed carcass counts (×) compared with posterior medians (dots) and 90% credible intervals (bars) as well as 50% intervals (inner ticks), based on ASF-positive wild boar carcasses found in the Kampinoski National Park during the years 2017–2021.

**Figure 6:**
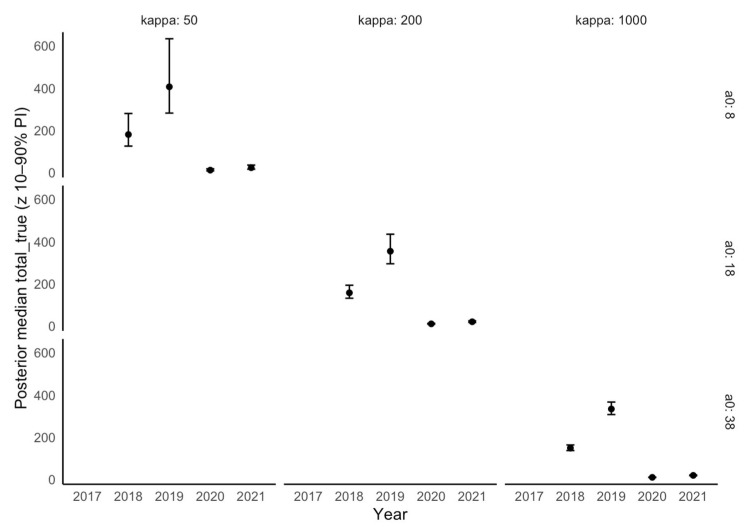
Robustness of posterior estimates of total wild boar mortality in Kampinoski National Park during the years 2017–2021) under alternative prior assumptions for carcass detectability (a_0_) and age-class variability (κ). (Dots—posterior medians; bars—10–90% credible intervals).

Regarding the effectiveness of wild boar carcass searches and the assessment of the actual temporal dynamics of ASF in the KNP (Figure 5), the sensitivity curves (Figure 5A) show the expected monotonic pattern: as the assumed detectability of carcasses decreases, the estimated total mortality increases. Bayesian analysis (Figure 5B) also produced estimates that clearly exceeded the empirical data: for 2018, the posterior median was approximately 157 deaths (90% CI: 125–207) compared with 63 observed, and for 2019, approximately 353 (281–460) compared with 266 observed. The posterior distributions were unimodal but broad, reflecting genuine uncertainty related to detection, while consistently indicating underestimation of total mortality. The direct comparison (Figure 5C) makes this bias explicit—posterior medians with 90% credible intervals are positioned above the empirical counts—whereas in 2020–2021, the posteriors concentrate around low values, consistent with the limited number of detected cases. These findings are further supported by robustness analyses, which confirm that the observed underestimation of total mortality is not an artifact of prior assumptions but a consistent feature of the data across alternative model parameterizations (Figure 6).

As in other regions of Poland, the spread of ASF in KNP followed a pattern from east to west. In 2017, the first cases of dead wild boars were discovered in the eastern part of the park near the densely populated suburbs of Warsaw. The carcasses were found along two main corridors. One was a natural ecological corridor along the Vistula River. In the southern section, wild boars were found in a narrow strip encompassing dense urban development and adjacent agricultural fields along a main road connecting numerous towns with Warsaw. This road is heavily traveled and is used by tens of thousands of vehicles every day, as it is an important commuter route for people working in the capital. In 2017, all dead wild boars were found on the periphery of the park. Of the 1275 wild boars culled in the park and its buffer zone that year, none tested positive for ASF.

**Figure 7:**
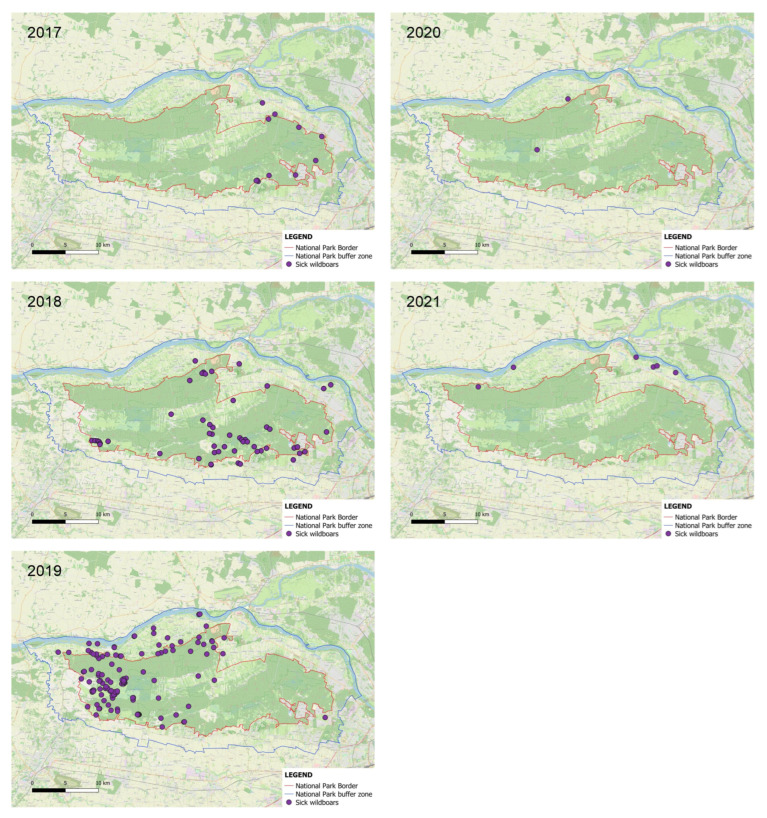
Spatial distribution of ASF-positive wild boars detected during carcass searches and culling operations in Kampinoski National Park in the years 2017–2021 (source: OpenStreetMap—https://opendatacommons.org/licenses/odbl/, accessed on 15 April 2025).

In 2018, ASF-positive wild boar carcasses were found throughout the eastern part of the park, mainly at the edge of forests and in transition zones between forests and cultivated fields. In addition, a significant cluster of seven ASF cases (about 7% of all wild boar deaths that year) was found in the westernmost part of the park near the tourist attraction Żelazowa Wola, the birthplace of Fryderyk Chopin. Of the 450 wild boars culled that year, three tested positive—two in the northern periphery and one in the southern. A smaller cluster (four cases, 4% of all carcasses) was detected inside the park near a settlement on the north–south road running through the park. In 2019, the disease spread to the western and northwestern parts of the park. Animal carcasses were again found both in these areas and along the ecological corridor along the Vistula River. Two ASF-positive wild boars were found on a large river island, Kępa Śladowska. Another infected wild boar, one of a total of seven ASF cases culled, was found on an island in the eastern part of the park. The remaining five infected animals were culled in areas that matched the locations of previous carcasses during routine examinations. In 2020, the epidemic appeared to have peaked. Of the 182 wild boars culled that year, only two were infected: one from the Vistula ecological corridor and another from the central part of the park. No ASF-positive carcasses were detected in the KNP that year. However, new ASF cases were reported in 2021. Although no carcasses were found, six out of 252 culled boars tested positive. All came from the Vistula corridor in the northern part of the park (Figure 7).

**Figure 8:**
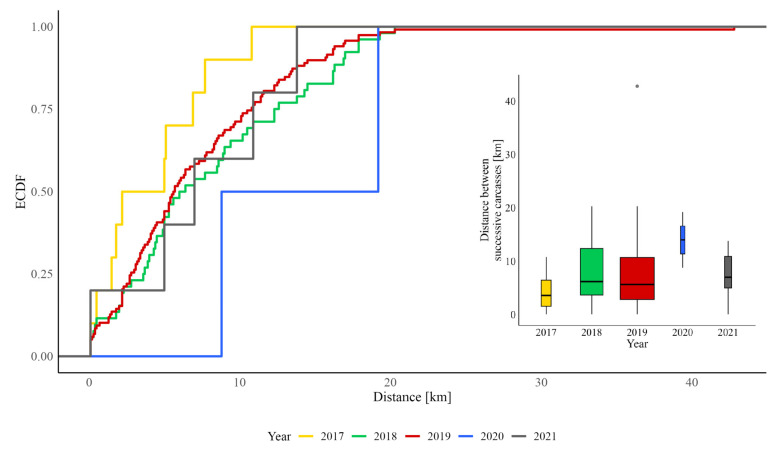
Empirical cumulative distribution functions (ECDF) of linear distances between ASF-positive wild boar carcasses and the characteristics of distances (min, Q25, median, Q75, max, shown on the boxplot) between consecutively detected carcasses in the years 2017–2021 in the Kampinoski National Park.

Throughout the epidemic, the disease spread in a patchy pattern—dead wild boars were found in groups at relatively short distances from each other. The mean distance between consecutive ASF cases during the epidemic was 7.3 km (SD = 5.94). Distances between consecutively found carcasses ranged from 0.1 km to 42.8 km, with a mean of 7.35 km (SD ≈ 5.9). The Kruskal–Wallis test showed no statistically significant differences among years (H = 6.41, *p* = 0.171), and the associated effect size was very small (ε^2^ = 0.013, 95% CI [–0.017, 0.051]). Pairwise Wilcoxon contrasts revealed no significant differences between pairs after *p*-value correction. However, Cliff’s delta estimates suggested moderate to large but imprecise effects (e.g., δ ≈ −0.90 for 2017–2020, 95% CI [−0.99, −0.44]), indicating potential biological but statistically uncertain variation. Visual exploration showed short inter-carcass distances in 2017, expanding in 2018–2019 during epidemic spread, and longer, more dispersed distances in 2020 when only a few isolated cases were found. In 2021, median distances returned toward earlier values, but the sample size was low. These patterns are consistent with an initial local outbreak, followed by spatial expansion and eventual fragmentation of infections (Figure 8).

## 4. Discussion

Shortly after the confirmation of the first case of ASF in Poland—in spring 2014, in a dead wild boar found near the eastern border of the country, about 300 km from KNP—it became clear that the disease would not remain local and spatially confined, unlike the situation in the Czech Republic, Hungary, or Belgium [7,9]. The spread of ASF in Poland soon took the form of a front several hundred kilometers long, moving westward and reaching the Vistula Valley in 2016 [39]. Thus, the appearance of the disease in KNP was only a matter of time. The first case of ASF in the park was recorded on 22 November 2017, followed shortly by additional cases. Between 2017 and 2021, the spread of the disease in KNP was rapid, peaking in 2018–2019. In KNP, as in other parts of the country, ASF spread mainly from east to west. This was facilitated by the compact structure of the forest complex, the absence of natural barriers, and the presence of an ecological corridor along the Vistula River. Humans may also have represented an additional vector in the case of KNP, as the “southern entry route” of the disease into the park—indicated by the locations of carcasses found in 2017—overlapped with densely built-up areas and major transport corridors. The possibility that humans act as a potential factor introducing the ASF virus into the environment (even though there is no evidence confirming direct transmission from humans to wild boar) is supported by studies from other European countries where ASF emerged far—sometimes several hundred kilometers—from previously known outbreak areas [7,8,9]. Although pre-emptive culling was undertaken before virus entry, these observations clearly suggest that it could not prevent introduction, and its role should therefore be interpreted primarily as mitigating subsequent spread rather than serving as a barrier to translocation.

National parks play an important ecological and social role—they protect natural heritage while remaining open to the public, providing space for recreation, education, and contact with nature [40,41,42]. However, this openness entails risks associated with human presence, including the potential transmission of pathogens. Intensive recreational use may lead to soil degradation [43], deterioration of water quality [44,45], and changes in the structure and composition of vegetation [5]. The presence of tourists also affects fauna—modifying animal behavior and causing other disturbances [46,47,48]. Moreover, recreation is considered one of the main pathways for the introduction of alien species into protected areas [49,50,51]. In this context, the risk of transmission of infectious diseases such as ASF becomes particularly significant. Although national parks in Poland cover only 1% of the country’s territory, they attract up to 30% of the total number of tourists [52], which in the case of KNP means as many as 1.5 million visitors annually. Tourists constitute a risk factor that is difficult to monitor—their mobility, random choice of routes, and wide travel range render both the direction and scale of transmission virtually unpredictable. The problem is exacerbated by the low level of visitor awareness regarding biosecurity and the lack of effective educational measures targeted at this group. While information campaigns during the ASF epidemic focused on foresters, farmers, and hunters, experiences from the Czech Republic and Hungary have shown that individuals not associated with these sectors could also serve as vectors of the virus [53,54,55]. Overall, human presence in national parks substantially increases the risk of ASF transmission and constitutes a strong rationale for implementing decisive management measures. It is also worth noting that, apart from general public advisories, no formalized biosecurity procedures for tourists existed in KNP prior to ASF detection, and measures directed at volunteer hunters were implemented only after the onset of the epidemic. This context further supports the plausibility of human-mediated introduction.

A major challenge in ASF control in large, protected areas is the technical and organizational constraints resulting from the specific functioning of national parks, which are not staffed or equipped to handle sudden and severe events such as natural disasters or epidemics of infectious diseases. In such cases, close cooperation between parks and relevant state services is considered necessary. Regarding ASF, KNP employs a relatively small number of field staff, only some of whom are authorized to use firearms, which in practice made intensive wild boar population control impossible. Therefore, it was necessary to engage volunteer hunters to support culling operations, while local fire brigades assisted in searching for carcasses. The experience of KNP demonstrates and confirms that effective ASF management requires cooperation between national parks and a wide range of entities, both professional and voluntary [56,57].

Although it is not possible—even in principle—to reconstruct the exact course of the disease, including its mortality, rate, and directions of ASF spread in the absence of preventive actions, it appears that wild boar population reduction played a central role in limiting the scale of the epidemic in KNP. Importantly, most culling occurred before ASF reached the park, suggesting that population decline had already been substantially advanced by management actions prior to the onset of disease-driven mortality. Although the sharp decline recorded in 2018–2019 coincided with both the peak of ASF mortality and the final period of intensive culling, the relative contribution of hunting versus disease cannot be quantified due to uncertainties in population size. This limitation should be explicitly acknowledged in any assessment of the effectiveness of depopulation measures. The absence of a reliable and rapidly applicable method for estimating wild boar abundance—especially under conditions of dynamic population change—remains an objective challenge, well recognized by both wildlife managers and the scientific community [56,57]. This is illustrated, among other evidence, by data from KNP, which show no clear population response to very intensive culling that in 2016–2017—immediately preceding the appearance of ASF in the park—exceeded the estimated spring population size by more than 100%. Notably, the effects of this intensive culling became apparent only two years later, in the spring of 2018, when a marked reduction in wild boar numbers was finally detected. These difficulties are further supported by data from Roztocze National Park, where between 2003 and 2021, five ungulate surveys conducted using two independent methods (drive counts and the pellet count method) produced wild boar estimates ranging from several dozen individuals to nearly 2000, reinforcing the widely accepted view that this species is among the most difficult to assess reliably [58]. Regardless of the discussion as to whether around 200 wild boars were indeed present in KNP in 2018, it can be assumed with high probability that at the time of ASF introduction, the population density of this species was at its lowest compared with previous years. It was precisely this low density that contributed to limiting the epidemic to just four years, with a two-year peak in infections.

Analysis of the age structure of dead and culled wild boars showed that the effectiveness of carcass searches—although described in the literature as one of the most efficient methods of limiting virus spread [59]—was in reality limited and probably significantly underestimated. Assuming a detectability level of 0.8—an optimistic scenario for effective removal of infected wild boar carcasses from the environment—resulted in an estimated total mortality approximately 30–60% higher than the number of observed carcasses. The imperfection of carcass searches is further confirmed by studies by Depner et al. (2017) [60] and Arias et al. (2018) [61], indicating that locating all dead individuals in large forest complexes is practically impossible. This limitation is crucial when interpreting the effectiveness of management actions, as unidentified carcasses may continue to serve as infection sources despite ongoing culling.

The decision on intensive wild boar population reduction in KNP, made with appropriate advance planning, must be regarded as both justified and effective. By lowering the number of animals, the park—despite strong anthropogenic pressure due to the proximity of the Warsaw metropolitan area and high tourist numbers—did not become a hotspot of ASF transmission to other parts of central Poland; indeed, no cases of the disease were recorded in areas west of KNP. Kampinoski National Park, however, was not the only one where reduction measures targeting wild boar populations were implemented. While in 2000–2013 wild boar culling in Polish national parks was carried out mainly to limit agricultural damage and involved between 500 individuals (in 2000) and 951 (in 2013), after the appearance of ASF in Poland the scale of these measures clearly increased—from 1189 wild boars culled in 2014 to 3076 in 2017 [62]. Unlike the national parks in the eastern part of the country, which fell within the ASF range and faced the challenge of population reduction under conditions of organizational pressure and disease presence, KNP was the only park where reduction culling was undertaken with considerable advance planning. This made it possible to refine the system of culling operations, manage the carcasses of both culled and later recovered dead individuals, and efficiently organize the submission of veterinary samples from a large number of animals distributed across several hundred square kilometers.

Depopulation, as a method of limiting the spread of ASF, although accepted by agricultural and veterinary communities [63,64], is not regarded as an effective remedy for this disease by part of the scientific community. The doubts concern primarily ethical issues, such as the mass culling of wild boars regardless of their health status, the implementation of culling in ASF-free areas despite the low probability of virus introduction, or the elimination of individuals of all ages and at all times of the year, including during pregnancy, reproduction, and parental care [65]. Ethical arguments, often raised by the public, are understandable and underscore the need for complementary educational measures, which should provide information on the methods and objectives of depopulation. Substantive concerns were also raised in studies conducted in the Białowieża Forest [66]. The authors, using camera trap recordings, compared data from two adjacent areas without migration barriers: a relatively small reserve (ca. 10 km × 10 km) with homogeneous habitats, largely composed of old stands, and the surrounding forest, more than five times larger, managed for timber production and subject to intensive hunting. Managed forests were characterized by a mosaic structure and direct proximity to agricultural fields, which further increased their attractiveness as foraging grounds. Considering the average daily range of wild boar movements (ca. 9 km, according to the authors), it is difficult to assume that the analyzed reserve could serve as a permanent refuge, and thus as a true control area relative to the hunted managed forest. Documented ungulate migrations between protected and managed areas [67,68], as well as results indicating seasonal fluctuations in density [69], further support these limitations. Consequently, the decrease in wild boar density observed in the reserve may have been largely the result of compensatory migration [70,71,72]. From this perspective, intensive culling may reduce numbers not only within hunted areas but also in neighboring small fragments of protected forests, which, in terms of limiting infectious disease transmission, should be considered a desirable effect. At the same time, it should be emphasized that the example of the Białowieża Forest is not fully comparable to the situation in Kampinos National Park, which is a large, compact, and isolated forest complex—here, effective population reduction could have been achieved only through measures carried out within the Park itself. Nevertheless, the interpretation of effectiveness must remain cautious given the uncertainty of population size estimates and the unquantified contribution of ASF-induced mortality. Since 2022, no new ASF cases have been recorded; however, reliable monitoring of the population’s sanitary status and efficient information flow remain crucial, enabling rapid and effective action in the future.

Due to the high reproductive potential of wild boar, which allows for a doubling of numbers after a single breeding season [73,74], and favorable environmental conditions providing abundant food, sufficient cover, and mild winters, wild boars rapidly rebuild their populations. According to official statistics, in 2023 the wild boar population in KNP reached approximately 750 individuals [21], which—regardless of the accuracy of the estimates—represents a level nearly three times higher than during the peak of reduction in 2018. Since 2022, no new ASF cases have been recorded; however, reliable monitoring of the population’s sanitary status and efficient information flow remain crucial, enabling rapid and effective action in the future.

## 5. Conclusions

The early decision to intensify wild boar culling in KNP led to a substantial reduction in the local population before ASF reached its peak. Although this intervention could not prevent the initial introduction of the virus, it likely helped limit both the spatial spread and overall impact of the epidemic within the Park.

Depopulation was not intended to block ASF incursion—an unrealistic goal given the epidemiological nature of the disease—but to ensure that any introduction would occur under conditions of reduced host density. This strategy appears to have mitigated the severity of the outbreak, especially considering the high lethality of ASF and the absence of treatment options.

Age-structure analyses of dead and culled wild boar indicate that actual ASF-related mortality may have been 30–60% higher than the number of laboratory-confirmed cases. This discrepancy underscores the challenges of wildlife disease surveillance and highlights the importance of considering detection bias when evaluating control effectiveness.

Although humans do not transmit ASF biologically, they may act as mechanical vectors. In heavily visited protected areas such as KNP, this pathway represents a significant risk. Enhancing visitor biosecurity awareness, along with targeted protocols for field personnel and volunteer hunters, should therefore be central to preparedness planning.

Organizational and staffing limitations can hinder both disease monitoring and the implementation of effective control measures in national parks. Strengthened cooperation between park authorities, local administrations, and hunting communities—supported by clear operational procedures and adequate resources for surveillance and carcass removal—is essential for improving outbreak response capacity.

While population reduction remains a controversial management option, it may serve as a useful component of an integrated response under specific high-risk conditions. Its application in protected areas, however, requires transparent justification, active stakeholder engagement, and measures to address animal welfare and societal concerns.

## Figures and Tables

**Table 1:** Age structure of wild boar populations free from ASF from Kampinoski National Park and other locations in Poland.

Location	Study	Proportion of Age Classes [%]		
		Piglets	**Yearlings**	**Adults**
KNP 1967	Andrzejewski & Jezierski 1978 [24]	52.5	30.3	17.2
KNP 1968		65.2	15.9	18.8
KNP 1969		40.5	33.1	26.4
KNP 1970		43.6	23.1	33.3
Zielonka	Fruziński 1992 [25]	49.0	34.9	16.1
Czempiń		45.0	32.0	23.0
Dolnośląskie—observations	Merta et al. 2005 [26]	42.7	33.4	23.9
Dolnośląskie—hunting		38.6	34.3	27.1
Śląskie—observations		58.7	22.8	18.5
Śląskie—hunting		53.4	24.1	22.5
MEAN (±SD)		48.9 (8.52)	28.4 (6.45)	22.7 (5.31)

**Table 2:** Prediction of the number of dead wild boars based on a comparison of the age structure of dead wild boars found in Kampinoski National Park in 2018 and 2019 with the age structure of wild boars from other studies conducted in Poland.

	Wild Boars with ASF Found and Shot in 2018 and 2019		Age Structure of Wild Boars from KNP and Other Sites in Poland	Estimated Actual Number of Wild Boar Carcasses	Difference Between Estimated and Detected Carcasses
	N	%	%	N	%
Year	2018				
Piglets	25	39.8	48.9	47	+88%
Yearlings	16	25.3	28.4	28	+75%
Adults	22	34.9	22.7	22	0
Total	63	100	100	97	+53%
Year	2019				
Piglets	136	51.1	48.9	153	+13%
Yearlings	59	22.2	28.4	89	+51%
Adults	71	26.7	22.7	71	0
Total	266	100	100	313	+18%

## Data Availability

Raw data can be provided upon a genuine request to the first author.
